# Association of coffee and tea intake with the risk of dementia: A prospective study

**DOI:** 10.1002/alz.086840

**Published:** 2025-01-09

**Authors:** Minqing Yan, Jie Shen, Yuhui Huang, Minyu Wu, Geng Zong, Yan Zheng, Changzheng Yuan

**Affiliations:** ^1^ Zhejiang University, Hangzhou, Zhejiang China; ^2^ School of Public Health, the Second Affiliated Hospital, Zhejiang University School of Medicine, Hangzhou, Zhejiang China; ^3^ Zhejiang University, Hangzhou China; ^4^ Zhejiang University, Hangzhou, Zhejiang province China; ^5^ Shanghai Institute of Nutrition and Health, Chinese Academy of Sciences, Shanghai China; ^6^ State Key Laboratory of Genetic Engineering, Human Phenome Institute, and School of Life Sciences, Fudan University, Shanghai China

## Abstract

**Background:**

The role of coffee and tea intake in dementia prevention remains inconclusive across studies.

**Method:**

We included 6,001 Health and Retirement Study (HRS) participants (67.5±10.0 years old, 59.0% female) without dementia at baseline and followed them until 2020. Coffee and tea intake (including caffeinated and decaffeinated coffee and tea) was assessed using a 163‐item semi‐quantitative food frequency questionnaire (FFQ) in 2013. Dementia diagnosis was ascertained using the Langa‐Weir Classification strategy. Cox proportional hazard models were used to evaluate the associations of coffee and tea intake with dementia.

**Result:**

During a median follow‐up of 7 years, 231 participants developed dementia excluding dementia cases within the first 2 years of follow‐up. Compared with participants who did not drink coffee, those drinking ≥2 cups/day were associated with lower risk of dementia (Hazards ratio [HR] = 0.62, 95% confidence interval [CI], 0.41, 0.94; P‐trend = 0.049). Associations were similar for caffeinated coffee (HR = 0.66, 95% CI = 0.46, 0.96) and decaffeinated coffee (HR = 0.49, 95% CI, 0.25, 0.99). Moderate intake of tea (>0 to < 2 cups/day compared with never) was also linked to a lower dementia risk (HR = 0.64, 95% CI, 0.47, 0.87 for >0 to <1 cup/day; HR = 0.55, 95% CI, 0.31, 0.98 for ≥1 to <2 cups/day). In addition, for caffeinated tea, drinking >0 to <1 cup per day was associated with a 43% lower risk of dementia (HR = 0.57, 95% CI, 0.43, 0.77), whereas no significant association were observed between decaffeinated tea and dementia.

**Conclusion:**

Higher consumption of coffee and moderate tea intake were associated with lower risk of dementia.

